# Antioxidant and In Vivo Hypoglycemic Activities of Ethanol Extract from the Leaves of *Engelhardia roxburghiana* Wall, a Comparative Study of the Extract and Astilbin

**DOI:** 10.3390/foods12050927

**Published:** 2023-02-22

**Authors:** Xiaoqiang Guo, Ting Zhou, Hongxia Xing, Yucheng Zhang, Jingmei Fang, Tairan Kang, Caimei Yao, Jun Yan, Yaxuan Huang, Qian Yao

**Affiliations:** 1 School of Pharmacy, Chengdu University, Chengdu 610106, China; 2 School of Food and Biological Engineering, Chengdu University, Chengdu 610106, China; 3 Zhanglan College, Chengdu University, Chengdu 610106, China

**Keywords:** leaves of *Engelhardtia roxburghiana* Wall, astilbin, antioxidant, α-glucosidase, hypoglycemic effects in vivo

## Abstract

The leaves of *Engelhardia roxburghiana* Wall (LERW) has been used as sweet tea in China throughout history. In this study, the ethanol extract of LERW (E-LERW) was prepared and the compositions were identified by HPLC-MS/MS. It indicates that astilbin was the predominant component in E-LERW. In addition, E-LERW was abundant in polyphenols. Compared to astilbin, E-LERW presented much more powerful antioxidant activity. The E-LERW also had stronger affinity with α-glucosidase and exerted more vigorous inhibitory effect on the enzyme. Alloxan-induced diabetic mice had significantly elevated glucose and lipid levels. Treatment with E-LERW at the medium dose (M) of 300 mg/kg could reduce the levels of glucose, TG, TC, and LDL by 16.64%, 12.87%, 32.70%, and 22.99%, respectively. In addition, E-LERW (M) decreased food intake, water intake, and excretion by 27.29%, 36.15%, and 30.93%, respectively. Moreover, E-LERW (M) therapy increased the mouse weight and insulin secretion by 25.30% and 494.52%. With respect to the astilbin control, E-LERW was more efficient in reducing the food and drink consumption and protecting pancreatic islet and body organs from alloxan-induced damage. The study demonstrates that E-LERW may be a promising functional ingredient for the adjuvant therapy of diabetes.

## 1. Introduction

Nowadays, diabetes is a high incidence disease seriously challenging human health. Diabetic patients occupy 10% of the world’s population. The complications of diabetes include renal injury, retinopathy, diabetic cataract, diabetic foot, coronary disease, and so on, which not only make the patients suffer great pain but also bring heavy economic burden on families and society. How to protect and treat diabetes has become a major concern in food and medicinal fields. Natural plants and their active ingredients exhibit multi-target, multi-pathway, and multi-directional hypoglycemic characteristics. Compared to chemical drugs, herbal medicines have mild and sustained effects with low toxicity. The multi-target property not only benefits glucose modulation, but also contributes to the alleviation of diabetic complications. A natural product with known hypoglycemic activity is becoming a promising alternative to the current drugs for diabetic therapy. *Engelhardtia roxburghiana* Wall (ERW) is a subtropical tree grown in the Guangdong, Guangxi, and Fujian provinces of China. The leaves of ERW (LERW) have been used as sweet tea in Chinese folk medicine to treat obesity, fever, and pain for a long time. Due to the abundance in flavonoids and phenols, LERW has multiple physiological activities, including inhibition of aldose reductase, bladder protection, as well as anticoagulant, hypolipidemic, and antioxidant activities [1]. Flavonoids such as astilbin, taxifolin, and engeletin are the main active ingredients responsible for the functions of LERW [2]. Among them, astilbin is the predominant component and is regarded as an important indicator to evaluate the quality of LERW.

As the major constituent of LERW, astilbin possesses versatile biological activities. Astilbin was able to inhibit the generation of superoxide anion and the peroxidation of microsomal lipid, thereby protecting red blood cells from oxidization and hemolysis [3]. Astilbin had an inhibitory effect on recombinant human aldose reductase and hampered the formation of advanced glycation end products, showing the potential in the prevention and treatment of diabetic syndrome [4]. Astilbin also presented its effects in the treatment of diabetes and related secondary complications [5], such as diabetic nephropathy. In addition, astilbin displayed the lipid-lowering capacity in rats by increasing the activity of lipoprotein lipase and promoting the lipolysis of rat fat pads [6].

Astilbin is the chief constituent of LERW. As the hypoglycemic effect of astilbin has been reported extensively, LERW is also assumed to possess hypoglycemic function. The safety and low toxicity of LERW have been well verified by its long-term usage as sweet tea, which makes it hold more immerse prosperity to serve as a healthcare product for the protection and treatment of diabetes. Astilbin, the primary active component, may be more efficient than the extract of LERW (E-LERW) in lowering glucose level. Nevertheless, there is another possibility that owing to the synergetic effect of other polyphenols present in LERW, the extract might possess stronger strength. It is important to clarify the activity difference between the purified component and E-LERW before the designing of LERW-based diabetic care products. This study aimed to compare the antioxidant activity of E-LERW and astilbin and evaluate their hypoglycemic effect via an in vitro α-glucosidase inhibitory test and an in vivo diabetic mouse model. HPLC coupled with tandem MS was used to determine and identify the polyphenols in E-LERW to illustrate the relationship between the hypoglycemic effect and the compositions of the extract.

## 2. Materials and Methods

### 2.1. Materials

LERW was purchased from Youluhuan Ecological Agriculture Co., Ltd. (Bozhou, China). 2,2-diphenyl-1-picrylhydrazyl (DPPH), 2,2’-azobis-3-ethylbenzothiazoline-6-sulfonate (ABTS), α-glucosidase, tannins, acarbose, and rutin were obtained from Shanghai Yuanye Biotechnology Co., Ltd. (Shanghai, China). p-nitrophenyl-β-D-galactopyranoside (pNPG) was obtained from Alfa Aesar Chemical Co., Ltd. (Shanghai, China). Astilbin with 98% purity was purchased from Priva Technology Development Co., Ltd. (Chengdu, China). Liposomes were prepared by our lab with the size of 131.84 ± 0.67 nm [7].

### 2.2. Preparation of E-LERW

The dried LERW was crushed, passed through a 60-mesh sieve, and extracted with 60% ethanol (*v/v*). The extraction was conducted with a MAR-3 microwave reactor (Shanghai Yuezong Instrument Company, Shanghai, China) under 56 °C for 67 s. The material-to-liquid ratio was 1:15. After the extraction, the sample was filtered, concentrated under reduced pressure, and finally freeze-dried to obtain the E-LERW [8].

### 2.3. Identification by HPLC-MS/MS

The extract was prepared into 1 mg/mL solution with 60% ethanol (*v/v*), filtered through a 0.22 μm microporous membrane, and separated on a Waters Acquity UHPLC BEH-C18 column (2.1 mm × 100 mm, 1.7 μm). The analysis was performed by an UHPLC system coupled with Xevo triple quadrupole electrospray tandem MS (Micromass Waters, Milford, MA, USA). The electrospray ionization source (ESI) was used for the determination of the components, and the full MS/dd-MS^2^ scan mode for qualitative and quantitative analysis. The sample of 10 μL was injected into the system. The mobile phase consisted of acetonitrile and 0.1% acetic acid (22:78, *v/v*) with the flow rate of 0.7 mL/min. The column temperature was 35 °C. Identification was performed by multiple reaction monitoring (MRM). The ions were detected in both positive and negative mode with *m/z* 100–1000. The other parameters of MS were set as follows: spray voltage 3.0 kV, S-lens voltage 50 V, capillary temperature 350 °C, and auxiliary gas heating temperature 350 °C [9]. In addition, the on-line UV spectrums of the components were obtained through diode array detection (DAD). The wavelength with maximum absorbance was determined.

### 2.4. Determination of Active Ingredients in E-LERW

#### 2.4.1. Total Flavonoids

The sample was prepared into 1 mg/mL with 60% ethanol. The content of total flavonoids was determined using the sodium nitrite–aluminum nitrate colorimetric method [10,11] and was expressed as mg rutin equivalent (mg RE)/g. In this study, the absorbance of the reference rutin changed linearly with the concentration in the range from 10 to 200 μg/mL. The regression equation was *A* = 11.094*C* − 0.0018 (*r*^2^ = 0.9993).

#### 2.4.2. Total Phenols

The total phenols in E-LERW were determined using the methods reported by Yao et al. and Dirar et al. [12,13] and were expressed as mg gallic acid (GA) equivalent (mg GE)/g. The absorbance of the reference GA was linear with the concentration ranging from 10 to 500 μg/mL. The regression equation was *Y* = 102.2*X* + 0.0616 (*r*^2^ = 0.9991).

#### 2.4.3. Astilbin

The sample was analyzed by a LD-20AD HPLC system (Shimadzu, Tokyo, Japan). The separation was performed on a SinoChrom ODS-BP column (4.6 mm × 150 mm, 5 μm). The detection conditions were the same as described in Section 2.3. The detection wavelength was 291 nm with the injection volume of 20 μL. In the range of 0.02 to 1.0 mg/mL, the peak area of astilbin was linear with the concentration. The regression equation was *Y* = 54756*X* − 255.86 (*r*^2^ = 0.9992).

### 2.5. Antioxidant Activity

#### 2.5.1. Scavenging DPPH Free Radicals

E-LERW and astilbin were prepared into a series of solutions, which contained astilbin from 0.2 to 1 mg/mL, respectively. The determination was carried out according to what Makgatho et al. reported [14]. Ascorbic acid was set as the positive control.

#### 2.5.2. Scavenging ABTS^+^ Radicals

The measurement was conducted following the method reported by Aruwa et al. [15].

#### 2.5.3. Ferric Reducing Activity of Power

The ferric reducing activity of power (FRAP) of E-LERW and astilbin were determined conforming to the method proposed by Hao et al. [16].

#### 2.5.4. Inhibition of Lipid Membrane Oxidation

The lyophilized liposomes were re-dispersed in deionized water, from which 0.5 mL was drawn out and blended with 0.5 mL of E-LERW or astilbin at different concentrations. The sample was incubated at 37 °C for 1 h. Subsequently, 1 mL of 1% thiobarbituric acid was added, boiled for 10 min, and cooled to room temperature. The solution was centrifuged at 1000 r/min for 10 min. The absorbance of the supernatant was measured at 532 nm (*A*). Meanwhile, the absorbance of blank control (*A*_0_) was determined using 0.5 mL deionized water in place of the sample. Tannic acid was set as the positive control. The inhibitory rate was calculated according to the following equation (Equation (1)) [17]:

Inhibitory rate = (*A*_0_ − *A*)/*A*_0_ × 100 (1)

### 2.6. Inhibitory Effect on α-Glucosidase

The inhibitory effect on α-glucosidase was examined according to the method described by Broholm et al. [18]. Briefly, the sample of 50 μL was blended with 50 μL α-glucosidase of 0.5 U/mL, and incubated under 37 °C for 30 min. Afterward, 1 mM substrate pNPG of 50 μL was added and reacted at 37 °C for another 30 min. The reaction was terminated by adding 0.2 M sodium carbonate of 50 μL. The absorbance at 405 nm was determined. In addition, using PBS to replace the enzyme, the background absorbance was measured in parallel. The inhibitory curve was constructed using the inhibitory rates versus astilbin concentrations. Acarbose was set as the positive control.

#### Kinetic Analysis on the Inhibition of α-Glucosidase

The concentration of α-glucosidase was fixed at 0.5 U/mL. The inhibitory velocity of E-LERW and astilbin on α-glucosidase was determined under different concentrations of substrate pNPG [19]. The double reciprocal curves were plotted based on the following Lineweaver–Burk equation:

(2) $$\frac{1}{v}=\frac{K_{m}}{v_{\max}}\left[1+\frac{\left[I\right]}{K_{i}}\right]\frac{1}{\left[S\right]}+\frac{1}{v_{\max}}\left[1+\frac{\left[I\right]}{\alpha K_{i}}\right]$$

and a secondary plot was constructed as Equation (3):

(3) $$\mathrm{Slope}=\frac{K_{m}}{V_{\max}}+\frac{K_{m}\left[I\right]}{V_{\max}K_{i}}$$

where *v* is the inhibitory velocity of the sample on α-glucosidase and [*I*] and [*S*] represent the concentration of inhibitor and substrate, respectively. *K_i_* and *K_m_* are the inhibition constant and Michaelis–Menten constant, respectively. α is a constant standing for the ratio of uncompetitive inhibition to competitive inhibition.

### 2.7. Hypoglycemic Activity In Vivo

#### 2.7.1. Animal Experiment Design

**Figure 1:**
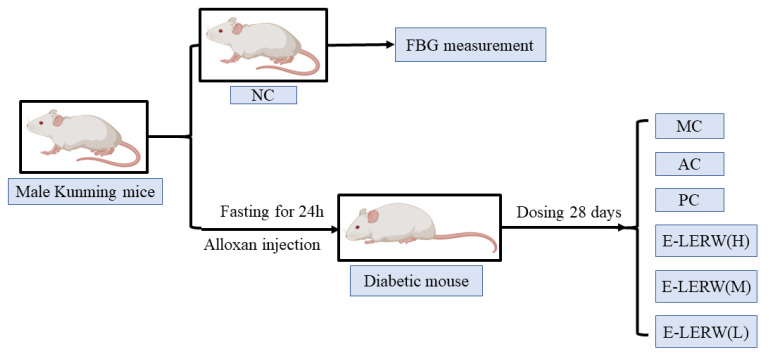
The scheme of animal experimental design. NC: The normal control of mice without alloxan injection. MC: The model control of diabetic mice without any treatment. PC: The positive control of mice treated with metformin. AC: The control of mice treated with astilbin (30 mg/kg). E-LERW (H), E-LERW (M), and E-LERW (L): The diabetic mice treated with ethanol extract of leaves of *Engelhardtia roxburghiana* Wall (E-LERW) at high dose (600 mg/kg), medium dose (300 mg/kg), and low dose (150 mg/kg), respectively. FBG: fasting blood glucose.

The animal experiment was approved by the Ethics Committee of Chengdu University, Chengdu, China (protocol number: CDPS 2020-122), and all procedures adhered to European Community Guidelines (86/609/EEC) for the Care and Use of Laboratory Animals. Male Kunming mice, weighing 18 to 22 g, were purchased from Chengdu Dashuo Experimental Animal Company (Chengdu, China). Before the experiment, all mice were allowed to adapt to the environment for 3 days. The mice in the normal control (NC) group were fasted but had free access to water for 12 h, and fasting blood glucose (FBG) was measured via the tail vein, which was used as the basic blood glucose level of normal mice. The rest of the mice were fasted for 24 h, followed by the intraperitoneal injection of alloxan at 200 mg/kg to develop a diabetic mouse model [20]. The fasting blood glucose was measured after 3 days. The mice with the blood glucose level over 11.1 mmol/L were diagnosed as diabetic mice and were randomly divided into 6 groups with 6 mice in each group. The groups include the model control of diabetes (MC); astilbin control (AC) with the dosage of 30 mg/kg; the positive control (PC) of metformin hydrochloride at the dose of 100 mg/kg; and E-LERW groups of high (H), medium (M) and low dose (L) at 600, 300, and 150 mg/kg, which were equivalent to the dose of 56.88, 28.44, and 14.22 mg astilbin/kg, respectively. The oral gavage was performed twice a day and consecutively lasted for 28 d [21]. The scheme of the experimental design was displayed in Figure 1.

#### 2.7.2. Oral Glucose Tolerance Test

At the final week of treatment, all mice were orally given a glucose solution of 1.5 g/kg after being fasted for 12 h [22]. The blood glucose level was measured every half hour. Oral glucose tolerance test was expressed as *AUC* in 2 h.

#### 2.7.3. Blood Sample Analysis

When the experiment was completed, the mice were sacrificed by breathing carbon dioxide. The mouse blood was collected in a tube pre-coated with heparin sodium and was centrifuged at 3000 r/min for 10 min. The supernatant serum was stored at −20 °C until measurement. The levels of insulin, triglyceride (TG), total cholesterol (TC), high density lipoprotein (HDL), and low-density lipoprotein (LDL) were measured by commercial ELISA kits (Nanjing Jiancheng Bioengineering Institute, Najing, China). All the determinations were carried out according to the instructions of the reagent kits.

#### 2.7.4. Organ Index

After the mice were sacrificed, the livers and kidneys were detached from the body, placed on filter paper to remove blood, and weighed, respectively. The weight ratios of organ to body (organ indexes) were calculated.

### 2.8. Data Analysis

All data are expressed as mean ± standard error. The diagrams were plotted using Origin 8.0 (OriginLab Corporation, Northampton, MA, USA). The difference between the data was evaluated by one-way analysis of variance (ANOVA) and Duncan’s test using SPSS version 10.0 software (IBM SPSS Inc., Chicago, IL, USA). The difference was considered statistically significant when *p* < 0.05.

## 3. Results

### 3.1. HPLC-MS/MS Analysis

**Figure 2:**
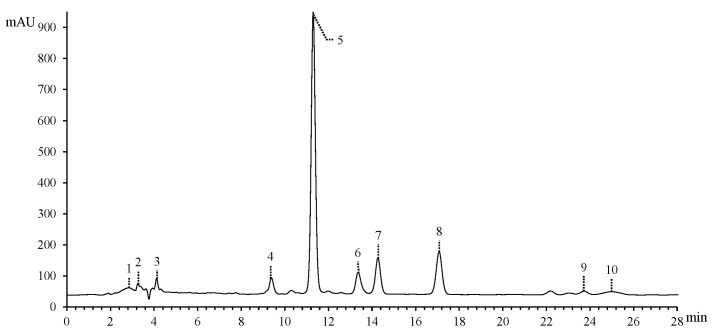
High-performance liquid chromatography with diode array detection (HPLC-DAD) chromatograms (291 nm) of E-LERW. **Compounds** are numbered as listed in Table 1.

The chromatogram and MS identification results of E-LERW are shown in Figure 2 and Table 1, respectively. A total of 10 components were identified with reference to the database of the instrument. α-Lactose was determined by the molecular ions of *m/z* 360.1497 (M+NH_4_)^+^ and 365.1050 (M+Na)^+^. The ion with m/z 145.0494 was assigned to hydroxypropyl pyran, which removed one water and formed the ion of m/z 127.0390. The ion further dissociated one propylene and yielded the ion with m/z 85.0289. Malic acid had the MS^2^ fragments of *m/z* 115.0023 (M-H-H_2_O, A) and 71.0125 (A-CO_2_). **Compound 3** displayed the ion of hydroxyl triazole ring with *m/z* 96.9682, which eliminated one water and produced the ion of *m/z* 78.9576. Quercetin presented the MS^2^ fragments of *m/z* 285.0385 (M+H-H_2_O, C), 257.0442 (C-CO, D), and 238.9389 (D-CO). In addition, the fragment of *m/z* 183.0285 was the reduced product from the flavone bone structure exclusive of catechol [23]. The ion 153.0181 was catechol lactone ring (C_6_H_2_(OH)_2_(OCOO)). Astilbin displayed the MS^2^ fragments of *m/z* 303.0607 (M-H-rhamnose, E) and 285.0400 (E-H_2_O). The ion of *m/z* 178.9975 was the oxidized flavone bone structure in the absence of catechol. This fragment removed one carbon oxide and formed the ion of *m/z* 151.0024. The compound engeletin and taxifolin also had the characteristic ions of 179 and 151, as astilbin presented. In addition, the peak of *m/z* 269.0452 in the spectrum of engeletin attributed to the detachment of one rhamnose from the parent molecule. Taxifolin presented the ions with *m/z* 285.0401 (M-H-H_2_O) [24] and 125.0231, which were assigned to pyrogallol [23]. The MS^2^ of citric acid included the ions of *m/z* 111.0074 and 87.0074, which was in accordance with what AliAbadi et al. reported [25]. **Compound 6** and **7** failed to be detected in the MS^2^ due to the weak fragment signals.

The flavonoid-like compounds from 4 to 9 had the maximum absorbance wavelength of around 290–295 nm [26]. Quercetin and maritimetin included the maximum wavelength of over 300 nm due to longer conjugate structure.

### 3.2. Determination of Active Components

The contents of astilbin, total flavonoids, and total phenols in E-LERW were 94.79 ± 2.49 mg/g, 153.42 ± 2.74 mg RE/g, and 255.74 ± 4.16 mg GE/g, respectively. It indicates that E-LERW is enriched in polyphenols.

### 3.3. Antioxidant Activity

**Figure 3:**
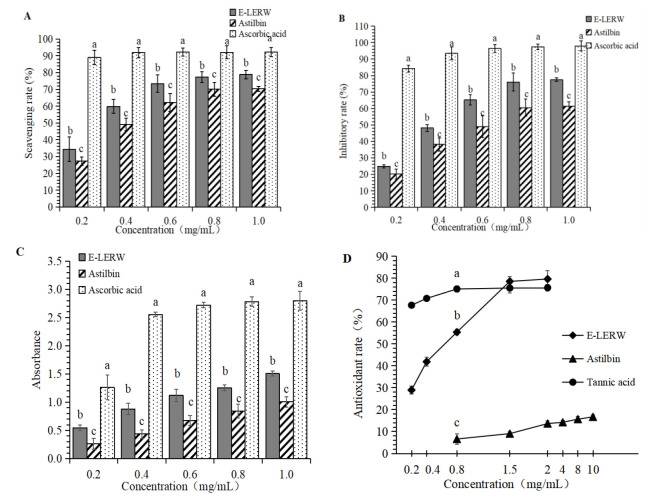
Antioxidant ability of E-LERW and astilbin: (**A**) Scavenging rate against DPPH radicals. (**B**) Scavenging rate against ABTS^+^ radicals. (**C**) FRAP. (**D**) Inhibitory rate against lipid membrane oxidation. All values were expressed as the mean ± SD from triplicate experiments. The different letters represent significant difference at *p* < 0.05 by ANOVA and Duncan’s test. FRAP: Ferric reducing antioxidant power. Other abbreviations are as in Figure 1.

The results of E-LERW in scavenging DPPH free radicals, ABTS+ free radicals, FRAP, and inhibition against lipid membrane oxidation are shown in Figure 3. The activity of both E-LERW and astilbin presented a concentration-dependent mode. The activity increased with the elevation of concentration. At different concentrations, the capacity of E-LERW in scavenging free radicals was significantly higher than that of astilbin (*p* < 0.05, Figure 2A,B). Meanwhile, E-LERW also exhibited much stronger FRAP over astilbin (*p* < 0.05, Figure 2C). E-LERW presented a more potent capacity in inhibiting the oxidation of lipid membrane as well (Figure 2D). When the concentration amounted to 2 mg/mL, E-LERW prevented 75% lipid membrane from oxidation while the inhibitory rate of astilbin was only less than 20% at the same concentration. The inhibitory effect of astilbin kept low even as the concentration reached 10 mg/mL. The control of ascorbic acid presented much stronger antioxidant activity over both astilbin and E-LERW in the examined concentration range (*p* < 0.01). When the concentration was below 1.5 mg/mL, tannic acid exhibited significantly higher inhibitory capacity against lipid membrane oxidation (*p* < 0.01).

### 3.4. Inhibitory Effects on α-Glucosidase

#### 3.4.1. Inhibition on α-Glucosidase

**Figure 4:**
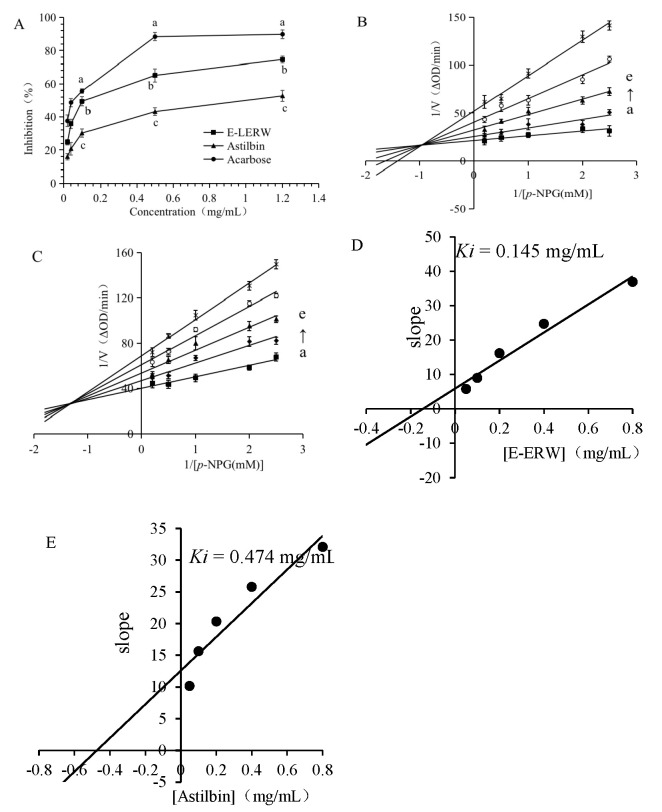
(**A**) The inhibitory activity of E-LERW and astilbin on α-glucosidase. (**B**) Lineweaver–Burk curve of E-LERW. (**C**) Lineweaver–Burk curve of astilbin. (**D**) The secondary plot of slope versus E-LERW concentration. (**E**) The secondary plot of slope versus astilbin concentration. (**A**)–(**E**): 0.05, 0.1, 0.2, 0.4, 0.8 mg/mL. All values were expressed as the mean ± SD of triplicate experiments. The different letters represent significant difference at *p* < 0.05 by ANOVA and Duncan’s test.

The inhibitory effect of E-LERW and astilbin on α-glucosidase is shown in Figure 4A. The inhibitory rates of both the samples and the control acarbose presented a concentration-dependent manner. The effect increased with the elevation of concentration. The inhibitory strength of E-LERW was remarkedly higher than that of astilbin in the examined concentration range (*p* < 0.05). Meanwhile, the control acarbose displayed much stronger inhibitory activity than E-LERW and astilbin (*p* < 0.05). The concentration with 50% inhibitory rate (IC_50_) of E-LERW, astilbin, and acarbose was 0.46 ± 0.09, 1.12 ± 0.17, and 0.19 ± 0.03 mg/mL, respectively.

#### 3.4.2. Inhibitory Kinetic Analysis

The Lineweaver–Burk curves of E-LERW and astilbin are shown in Figure 4B,C, respectively. The increase of the concentration accompanied with the elevation of the vertical axis intercept (1/*V_max_*), as well as the decrease of the net value of horizonal axis intercept, indicate that the interaction between the samples and α-glucosidase belonged to a mixed mode [19]. The secondary plot using slope-versus-inhibitor concentration was linear (Figure 4D,E), showing that both E-LERW and astilbin had a single inhibitory site on α-glucosidase. The calculated *Ki* of E-LERW and astilbin was 0.145 and 0.474 mg/mL, respectively.

### 3.5. Hypoglycemic Activity In Vivo

#### 3.5.1. Body Weight, Food Intake, Water Intake and Excretion

Table 2 shows the body weight, the amounts of excretion, and food and water consumption of mice in different groups. On the first day of alloxan injection, the diabetic mice had similar food intake to normal mice, but with more than threefold the water consumption and, as a result, over three times the excretion compared to the normal mice. This demonstrated a successful establishment of a diabetic mouse model. Though the body weights of mice in all groups increased after 28 d, the weights of the mice injected with alloxan were significantly lower than those in normal control (NC) group, who received no injection (*p* < 0.05). Nevertheless, compared to the model control (MC) group without any therapy, the groups with the treatment of metformin (PC), astilbin (AC), and E-LERW of high (H) and medium dosage (M) had the weight increment of 49%, 18%, 38%, and 25%, respectively, affirming the remedy effectiveness of metformin, astilbin, and E-LERW on diabetes. Though the weights of diabetic mice decreased, their food intake, water intake, and excretion increased dramatically (*p* < 0.01). The food and drink consumed by the mice in MC group were 1.8 and 6.3 times the amount consumed by normal mice. After the treatment of metformin, astilbin, and E-LERW at high (H), medium (M), and low dosage (L), the food intake diminished to 1.13, 1.38, 1.18, 1.31, and 1.74 times the normal intake, respectively. The drinking dropped to 2.79, 4.38, 3.33, 4.02, and 6.16 times normal drinking, respectively. The excretion of MC mice was seven times that of normal mice. Through treatment with different samples, the excretion reduced to 3.21, 6.30, 3.93, 5.04, and 7.09 times the normal amount, respectively. The results show that metformin (PC) has the most powerful therapeutic effect, followed by E-LERW (H) and (M). Astilbin (AC) and E-LERW (L) have weak activity in alleviating the symptoms triggered by a high glucose level.

#### 3.5.2. Fasting Blood Glucose and Insulin

**Figure 5:**
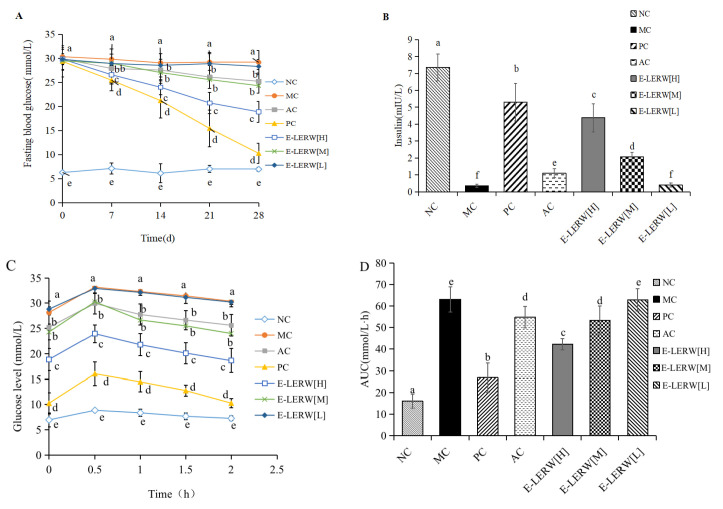
Effects of astilbin and E-LERW on the fasting blood glucose level (**A**), insulin secretion (**B**), oral glucose tolerance test (**C**), and *AUC* (**D**). All values were expressed as the mean ± SD (*n* = 6). Data analysis was performed by one-way ANOVA and Duncan’s test. Different letters indicate significant differences among groups (*p* < 0.05).

Figure 5A shows the fasting blood glucose (FBG) levels of mice receiving different treatments during 28 d. As time progressed, the MC and the group fed with E-LERW (L) maintained high and invariable glucose levels. Other diabetic mice treated with different samples had a gradually declining FBG. On day 28 of the therapy, the FBG of the mice receiving metformin, astilbin, and E-LERW (H) and (M) was reduced to 35%, 87%, 65%, and 83% level of MC group, respectively. Metformin again presented the strongest hypoglycemic activity. Astilbin and E-LERW exhibited moderate strength. E-LERW (M) included approximately 10% astilbin, which was equivalent to the AC group.

Figure 5B indicates that the injection of alloxan severely damaged the function of islet. The insulin level of MC mice was only 4.7% that of normal mice. Under the treatment of metformin, astilbin, and E-LERW (H, M and L), insulin secretion was restored to 72.0%, 15.0%, 59.5%, 28.0%, and 5.4% normal level, implying that astilbin and E-LERW helped to restore the damaged islets.

#### 3.5.3. Oral Glucose Tolerance Test

Oral glucose tolerance and the corresponding area under the curve (*AUC*) of each group are displayed in Figure 4C,D, respectively. The results show that the glucose peak values of all groups were reached in 30 min after the oral administration of glucose, followed by a gradual decrease. The glucose peak concentration of MC was increased to 3.74 times that of normal mice. After the treatment of metformin, astilbin, and E-LERW (H, M, and L) for 28 d, the peak level was reduced to 1.81, 3.38, 2.70, 3.41, and 3.71 times the normal level, showing the therapeutic effect of metformin, astilbin, and E-LERW in improving the oral glucose tolerance of diabetic mice.

*AUC* is another indicator to assess the oral glucose tolerate. The *AUC* of MC was 3.94 times that of normal mice, verifying the alloxan-induced impairment of glucose tolerate. The value was reduced to 1.67 and 3.43 times the normal level after the remedy of metformin and astilbin, respectively. E-LERW (H, M, and L) decreased the *AUC* to 2.64, 3.33, and 3.93 times the normal value. The trend was similar to the effects of various samples in diminishing glucose peak concentration. Meanwhile, the hypoglycemic activity of E-LERW (M) was consistent with that of astilbin control.

#### 3.5.4. Blood Lipid Analysis

Patients with diabetes and prediabetes are always at increased risk of dyslipidemia and cardiovascular disease [27]. As shown in Table 3, the injection of alloxan also significantly increased the levels of TG, TC, and LDL, and remarkedly reduced the concentration of HDL in MC mice (*p* < 0.01). The administration of various samples decreased the lipid levels and boosted HDL concentration to different degrees. The lipid lowering strength was metformin > E-LERW (H) > astilbin and E-LERW (M) > E-LERW (L) (*p* < 0.05). E-LERW (M) presented stronger activity in reducing TC and LDL with respect to astilbin, but the difference was not significant (*p* > 0.05).

#### 3.5.5. Effects of E-LERW on Organ Indexes of Liver and Kidney

The status of high glucose level impairs livers and kidneys as well. The organ indexes of mice in each group are shown in Table 4. Compared to the normal mice, the liver index of the MC group increased 33%. Other groups such as metformin, astilbin, and E-LERW (H, M, and L) elevated 8%, 27%, 12%, 21%, and 34%, respectively. The kidney index of the MC group increased 67%, while that of the treatment groups rose 8%, 51%, 27%, 44%, and 65%, respectively. It indicates that diabetes exerts a more detrimental impact on kidneys. E-LERW has the function of preventing liver and kidney swelling. The medium dose exhibited stronger capacity than the purified compound astilbin in protecting the organs.

## 4. Discussion

Compared to astilbin, the LERW presented much stronger antioxidant as well as α-glucosidase-inhibitory activity in vitro. Perez-Najera et al. obtained astilbin enriched extract from Smilax aristolochiifolia Root with astilbin at 48.76 mg/g [28]. The inhibitory rate of the extract against α-glucosidase was lower than 10%. The vigorous strength of E-LERW may originate from the integrative effect from both astilbin and other flavonoids present in LERW, such as quercetin and engeletin. Moreover, in the inhibitory kinetic test, the *Ki* of astilbin was 3.27 times that of LERW, implying that the affinity between the enzyme and LERW was much stronger than astilbin.

In the animal experiment, E-LERW significantly lowered blood glucose levels of mice triggered by alloxan. The group of E-LERW (M) had a similar content of astilbin to the group of astilbin control (AC). Though E-LERW exhibited much stronger antioxidant and glucosidase-inhibitory effects over astilbin, compared to AC, E-LERW (M) did not display more powerful effect in lowering fasting glucose level or enhancing oral glucose tolerance. The possible reason is that the hypoglycemic process involves various complex mechanisms—for example, decreasing glucose absorption from small intestine, hindering glucose production in vivo, prompting glucose uptake by tissues, enhancing glucose clearance from body, and so on [29]. Recent studies found that DNA methylation, histone modification, and non-coding RNA expressing also contribute to the pathogenesis of diabetes [30]. Inhibition on α-glucosidase only means the yield of glucose is reduced and glucose absorption is slowed down. It indicates that compound astilbin is the major component responsible for the hypoglycemic function of E-LERW.

Though the glucose level of the mice treated with E-LERW (M) was similar to those with astilbin, E-LERW (M) group had significantly higher insulin concentration than AC group, implying the protective capacity of flavonoids and polyphenols present in the extract on the islet β-cells. Flavonoids were able to increase the numbers of islets and β-cells, restore the pancreatic tissues impaired by alloxan, decrease β-cell apoptosis, and activate insulin receptors, which resulted in the increase of insulin secretion [31]. The underlying mechanisms for flavonoids and polyphenols to preserve β-cells include the blocking of NF-kappa B signaling, activation of the PI3K/Akt pathway, as well as the release decrease of nitric oxide (NO) and reactive oxygen species (ROS) [32].

Alloxan injections led to hyperglycemia accompanied with significant weight loss, while food intake, water intake, and excretion amount increased dramatically (Table 2). The phenomena were in accordance with what Leme et al. reported [33]. Administration of astilbin and E-LERW (H) and (M) significantly alleviated diabetes-induced weight loss, food intake, water intake, and excretion amount (*p* < 0.05). Compared to astilbin, E-LERW (M) reduced water intake and excretion more efficiently (*p* < 0.05). Hyperglycemia also damaged the liver and kidney and made the two organs swell. E-LERW protected the liver and kidney by remarkedly diminishing the organ indexes. The group with E-LERW (M) had lower organ indexes of liver and kidney compared to the astilbin group, exhibiting more potent protective power on organs. This function is associated with the strong antioxidant activity of E-LERW [34]. Hyperglycemia mellitus is related to high yield of ROS, which may cause DNA oxidation. High levels of genomic damage led to liver and renal failure [35,36]. Antioxidant phytochemicals such as phenolic compounds and flavonoids help to scavenge ROS and protect the organs from radical related impairment [34]. The antioxidant components could also enhance the activity of antioxidant enzymes such as glutathione peroxidase and catalase [37] and lower the elevated levels of malondialdehyde (MDA) and NO in streptozotocin (STZ)-induced diabetic rats [38]. In addition, polyphenols and flavonoids were able to hinder the activity change of hepatic enzymes, for example, alanine aminotransferase (ALT), aspartate aminotransferase (AST) and lactate dehydrogenase (LDH), and attenuated the hepatic toxicity caused by STZ [39].

## 5. Conclusions

Astilbin was the principal component of E-LERW. Compared to astilbin, E-LERW presented significantly higher activity in scavenging radicals, FRAP, and inhibiting the oxidation of lipid membrane. E-LERW also displayed stronger affinity with α-glucosidase with more powerful inhibitory strength on the enzyme, which was evidenced by Lineweaver–Burk curves. After the alloxan injection, the plasma levels of FBG, oral glucose tolerance, TG, TC, and LDL of the mice increased to 4.18, 3.93, 2.04, 2.84, and 4.63 times the normal levels, respectively. Meanwhile, insulin secretion and HDL levels were reduced to 4.72% and 38.97% of normal mice. Alloxan also impaired the organs, causing the indexes of the liver and kidney to elevate 33% and 67%, respectively. Treatment with E-LERW (M) and (H) can efficiently lower the increased glucose and lipid levels induced by alloxan and boost the levels of insulin and HDL. In addition, E-LERW alleviated hyperglycemia-induced organ damage and decreased the liver and kidney indexes. Compared to astilbin control, E-LERW did not show more potent capacity in lowering glucose level and oral glucose tolerance, but presented a more efficient ability in preventing weight loss, reducing food intake, water intake, and excretion. Moreover, E-LERW was superior to astilbin in enhancing insulin secretion and protecting organs. The study indicates that E-LERW may be a promising functional ingredient in alleviating symptoms of diabetic patients.

## Figures and Tables

**Table 1:** Composition identifications by HPLC-MS/MS.

No.	RT (min)	Molecular Ion *m/z*	Molecular Weight	Fragment		λ_max_	Formula	Compound
				*m/z*	Abundance (%)			
**1**	2.84	360.1497 (M+NH_4_)^+^, 365.1050 (M+Na)^+^	342.1158	85.0289 127.0390 145.0494	100 65.63 68.75		C_12_H_22_O_11_	α-Lactose
**2**	3.26	133.0129 (M−H)^−^	134.0202	115.0023 71.0125	100 64.76		C_4_H_6_O_5_	Malic acid
**3**	4.42	277.0325 (M−H)^−^	278.0398	96.9682 78.9576	100 36.52		C_9_H_6_N_6_O_5_	3,5-Dinitro-n-(4H-1,2,4-triazole-4-yl)-benzamide
**4**	9.38	303.0497 (M+H)^+^	302.0426	303.0497 285.0385 257.0442 238.9389 183.0285	100 1.18 2.35 29.41 32.35	234 291 360	C_15_H_10_O_7_	Quercetin
**5**	11.30	449.1083 (M−H)^−^	450.1155	303.0607 285.0400 178.9975 151.0024	19.23 50.00 0.12 100	228 291	C_21_H_22_O_11_	Astilbin
**6**	13.36	465.1024 (M+H)^+^	464.0951			229 290	C_21_H_20_O_12_	Bractein
**7**	14.27	287.0548 (M+H)^+^	286.0473			295 338	C_15_H_10_O_6_	Maritimetin
**8**	17.08	433.1133 (M−H)^−^	434.1206	433.1133 269.0452 178.9975 152.0103	68.29 78.05 97.56 100	293	C_21_H_22_O_10_	Engeletin
**9**	23.73	303.0508 (M−H)^−^	304.0579	285.0401 125.0231	58.33 100	291	C_15_H_12_O_7_	Taxifolin
**10**	24.97	191.0188 (M−H)^−^	192.0260	111.0074 87.0074	100 52.17		C_6_H_8_O_7_	Citric acid

λ_max_ is the wavelength with the maximum absorbance in the UV spectrum, which was determined by DAD.

**Table 2:** Body weight, food intake, water intake, and excretion of mice treated with astilbin and E-LERW.

Group	Body Weight (g)		Food Intake (g)		Water Intake (mL)		Excretion (g)	
	1st Day	28th Day	1st Day	28th Day	1st Day	28th Day	1st Day	28th Day
NC	22.72 ± 0.69	41.08 ± 1.23 ^a^	5.76 ± 0.53	6.68 ± 0.28 ^a^	5.49 ± 0.36	6.42 ± 0.42 ^a^	5.38 ± 0.63	5.14 ± 0.57 ^a^
MC	22.83 ± 0.48	23.56 ± 0.41 ^e^	6.57 ± 0.26	12.02 ± 1.57 ^d^	18.12 ± 1.63	40.47 ± 2.36 ^f^	16.84 ± 1.58	36.03 ± 2.14 ^f^
PC	23.55 ± 0.61	35.13 ± 0.74 ^b^	6.42 ± 0.54	7.57 ± 0.83 ^b^	17.32 ± 1.34	17.89 ± 1.25 ^b^	16.80 ± 1.27	16.50 ± 1.16 ^b^
AC	23.41 ± 0.43	27.87 ± 0.37 ^d^	6.21 ± 0.68	9.24 ± 0.76 ^c^	19.77 ± 2.31	28.13 ± 1.57 ^e^	16.90 ± 1.93	32.37 ± 1.38 ^e^
E-LERW[H]	23.39 ± 0.57	32.47 ± 0.26 ^c^	6.31 ± 0.36	7.88 ± 0.33 ^b^	18.68 ± 0.93	21.38 ± 0.73 ^c^	17.37 ± 0.83	20.18 ± 1.11 ^c^
E-LERW[M]	23.36 ± 0.27	29.52 ± 0.52 ^d^	6.12 ± 0.72	8.74 ± 0.63 ^c^	19.83 ± 1.35	25.84 ± 1.24 ^d^	17.50 ± 0.92	28.49 ± 1.39 ^d^
E-LERW[L]	22.85 ± 0.36	23.07 ± 0.27 ^e^	6.27 ± 0.63	11.64 ± 1.14 ^d^	18.85 ± 0.83	39.56 ± 2.34 ^f^	18.23 ± 1.46	36.44 ± 2.12 ^f^

NC: The normal control of mice without alloxan injection. MC: The model control of diabetic mice without any treatment. PC: The positive control of mice treated with metformin. AC: The control of mice treated with astilbin (30 mg/kg). E-LERW [H], E-LERW [M], and E-LERW [L]: The diabetic mice treated with E-LERW at high dose (600 mg/kg), medium dose (300 mg/kg), and low dose (150 mg/kg), respectively. Data analysis was performed by one-way ANOVA and Duncan’s test. All values were expressed as the mean ± SD (*n* = 6). Different letters represent significant difference (*p* < 0.05).

**Table 3:** Lipid levels of mice treated with astilbin and E-LERW of different doses.

Group	TG (mmol/L)	TC (mmol/L)	HDL (mmol/L)	LDL (mmol/L)
NC	0.727 ± 0.13 ^a^	0.409 ± 0.09 ^a^	5.673 ± 1.21 ^a^	0.526 ± 0.14 ^a^
MC	1.484 ± 0.52 ^c^	1.162 ± 0.11 ^d^	2.211 ± 0.35 ^e^	2.436 ± 0.25 ^e^
PC	0.734 ± 0.23 ^a^	0.411 ± 0.17 ^a^	4.521 ± 0.56 ^b^	0.885 ± 0.22 ^b^
AC	1.174 ± 0.2 ^b^	0.848 ± 0.24 ^c^	2.982 ± 0.28 ^d^	2.045 ± 0.31 ^c^
E-LERW[H]	0.786 ± 0.15 ^a^	0.573 ± 0.16 ^b^	3.897 ± 0.51 ^c^	0.961 ± 0.13 ^b^
E-LERW[M]	1.293 ± 0.03 ^b^	0.782 ± 0.22 ^c^	2.794 ± 0.17 ^d^	1.876 ± 0.47 ^c^
E-LERW[L]	1.423 ± 0.21 ^c^	1.149 ± 0.12 ^d^	2.355 ± 0.33 ^e^	2.378 ± 0.36 ^e^

All values were expressed as the mean ± SD (*n* = 6) of triplicate experiments. Data analysis was performed by two-way ANOVA and Duncan’s test. Different letters mean significant difference (*p* < 0.05).

**Table 4:** The effect of astilbin and E-LERW on the organ indexes of diabetic mice.

Group	Liver (%)	Kidney (%)
NC	4.76 ± 0.34 ^a^	1.47 ± 0.25 ^a^
MC	6.33 ± 0.41 ^f^	2.45 ± 0.37 ^d^
PC	5.16 ± 0.18 ^b^	1.59 ± 0.48 ^a^
AC	6.06 ± 0.07 ^e^	2.22 ± 0.28 ^c^
E-LERW[H]	5.32 ± 0.16 ^c^	1.87 ± 0.23 ^b^
E-LERW[M]	5.78 ± 0.37 ^d^	2.11 ± 0.14 ^c^
E-LERW[L]	6.37 ± 0.73 ^f^	2.42 ± 0.21 ^d^

All values were expressed as the mean ± SD (*n* = 6) of triplicate experiments. Data analysis was performed by two-way ANOVA as well as Duncan’s test. Different letters mean significant difference (*p* < 0.05).

## Data Availability

The data used to support the findings of this study can be made available by the corresponding author upon request.
